# Administration of Bacterial Lipopolysaccharide during Early Postnatal Ontogenesis Induces Transient Impairment of Long-Term Synaptic Plasticity Associated with Behavioral Abnormalities in Young Rats

**DOI:** 10.3390/ph13030048

**Published:** 2020-03-18

**Authors:** Tatyana Y. Postnikova, Alexandra V. Griflyuk, Julia L. Ergina, Olga E. Zubareva, Aleksey V. Zaitsev

**Affiliations:** Sechenov Institute of Evolutionary Physiology and Biochemistry of RAS, 44, Toreza Prospekt, Saint Petersburg 194223, Russia; tapost2@mail.ru (T.Y.P.); griflyuk.al@mail.ru (A.V.G.); for.mail.ergin@gmail.com (J.L.E.); zubarevaoe@mail.ru (O.E.Z.)

**Keywords:** bacterial lipopolysaccharide, long-term potentiation, hippocampus, open field test, early life

## Abstract

Infectious diseases in early postnatal ontogenesis often result in cognitive impairments, particularly learning and memory. The essential foundation of learning and memory is long-term synaptic plasticity, which depends on N-methyl-D-aspartate (NMDA) receptors. In the present study, bacterial infection was modeled by treating rat pups with bacterial lipopolysaccharide (LPS, 25 µg/kg) three times, during either the first or the third week of life. These time points are critical for the maturation of NMDA receptors. We assessed the effects of LPS treatments on the properties of long-term potentiation (LTP) in the CA1 hippocampus of young (21–23 days) and adolescent (51–55 days) rats. LTP magnitude was found to be significantly reduced in both groups of young rats, which also exhibited investigative and motor behavior disturbances in the open field test. No changes were observed in the main characteristics of synaptic transmission, although the LTP induction mechanism was disturbed. In rats treated with LPS during the third week, the NMDA-dependent form of LTP was completely suppressed, and LTP switched to the Type 1 metabotropic glutamate receptor (mGluR1)-dependent form. These impairments of synaptic plasticity and behavior were temporary. In adolescent rats, no difference was observed in LTP properties between the control and experimental groups. Lastly, the investigative and motor behavior parameters in both groups of adult rats were similar.

## 1. Introduction

Infectious diseases in early postnatal ontogenesis often result in impairments of cognitive functions, especially learning and memory, with central nervous system infections resulting in severe impairments of these functions in children [1]. Experimental studies in rats have shown that even a single episode of inflammation during the neonatal period might alter the developmental trajectory of the maturing brain [2]. The administration of bacterial lipopolysaccharide (LPS) in different doses is commonly used as a model of bacterial infection [3]. By binding to toll-like receptor 4, LPS promotes pro-inflammatory gene expression in the cells of the immune and nervous systems, including the expression of cytokines such as interleukin-1β (IL-1β), interleukin 6 (IL-6), or tumor necrosis factor alpha (TNF-α), as well as the decreased expression of transforming growth factor beta (TGF-β), a cytokine with immunosuppressive and anti-inflammatory properties [4,5,6,7]. The administration of low doses of LPS in rats in early postnatal ontogenesis induced the accumulation of IL-6 in the juvenile period [8]; and later, in adulthood, the same doses resulted in impaired behavior in the fear conditioning test [2], and in the Morris water maze [9,10]. Similarly, the administration of pro-inflammatory cytokines, such as IL-1β or TNF-α, led to impairments in the performance of passive and active avoidance tasks or long-term increases in anxiety, in addition to changes in investigative behavior, in adolescent and adult rats [11,12].

The essential foundation of learning and memory is synaptic plasticity [13,14]. As such, a disturbance in its properties due to inflammation might cause cognitive impairments [8]. Synaptic plasticity refers to the ability of chemical synapses to change their strength as a result of previous synaptic activation [15]. The different patterns of neuronal activity can either increase or decrease synaptic efficacy. The long-term potentiation (LTP) of synaptic response can be induced in a variety of ways [16]; however, more physiologically relevant is a theta-burst stimulation (TBS) consisting of short bursts of 4–5 pulses at 100 Hz, with bursts repeated at 5 Hz (‘Θ’), and typically 10 bursts in one train [17,18].

The application of LPS in high doses in vivo and in vitro decreases the magnitude of LTP [4,19], most likely through an increase in pro-inflammatory cytokines [20]. It has been shown that LTP can either be promoted or prevented through the interference of TNF-α and IL-1β in the pathways controlling the molecular and structural synaptic changes indicative of LTP [20]. A significant increase in IL-1β levels inhibits synaptic strength and LTP in rat [21] and mouse [22] hippocampal slices. The induction of LTP in mice hippocampal slices was prevented by the application of IL-6 [23]. The effect of TNF-α on different forms of synaptic plasticity is rather complicated and depends on brain areas and experimental protocols [24,25,26,27]. In hippocampal slices, it has been shown that LTP is dose-dependently weakened by a long-lasting TNF-α application [28]. The reduction in the level of TGF-β expression due to the use of LPS may also impair LTP induction [29].

Today, it is evident that LTP can involve several mechanisms depending on the cell type, development stage, and conditions in which the synapses operate [14,18,30]. The postsynaptic Ca^2+^ influx is essential for the induction of LTP [31], and different forms of LTP are induced by spatially discrete Ca^2+^ sources [18,32,33]. One of the critical Ca^2+^-permeable receptors involved in hippocampal synaptic plasticity is the N-methyl-D-aspartate (NMDA) type of glutamate receptor [18,34]. The NMDA receptor (NMDAR) forms a heterotetramer with two obligatory GluN1 subunits and either two GluN2 subunits or a combination of GluN2 and GluN3 subunits [35]. There are four different GluN2 subunits (GluN2A-D), but receptors containing GluN2A and GluN2B subunits are predominant in the cortex and hippocampus [36]. The GluN2 subunits have different biophysical properties and roles in synaptic plasticity [37,38,39,40]. In the rat hippocampus, the expression of GluN2B is high at birth but decreases within the first two to three postnatal weeks; conversely, the expression of GluN2A increases with age, becoming more numerous than GluN2B in the hippocampus at the end of the third week of life [41]. It is assumed that inflammation occurring soon after birth can affect the maturation of synapses, and that the subunit composition of NMDARs can be disturbed [2], resulting in a disruption of synaptic plasticity.

The coexistence of distinct activity-dependent systems of synaptic plasticity based on discrete Ca^2+^ sources, such as NMDARs and metabotropic glutamate receptors (mGluRs), has been recently described in the same synapses [33]. Their relative roles in plasticity may change in some pathological conditions. For example, recently, we demonstrated a decrease in LTP magnitude due to transient switching between NMDA-dependent and mGluR1-dependent forms of LTP following pentylenetetrazole-induced status epilepticus in young rats [42]. Similar changes may occur after LPS administration. However, the exact mechanisms of LTP impairment have not yet been sufficiently studied. Thus, in the present study, we assessed the effects of low-dose LPS treatments at various times of early postnatal ontogenesis, before and during the maturation of NMDARs, on the properties of LTP in the CA1 hippocampus of young (21–23 days) and adolescent (51–55 days) rats. The behavior was assessed in young (23–25 days) and adult (3 months) rats.

## 2. Results

### 2.1. Hippocampal LTP in Young Rats is Weakened after the Administration of LPS in Early Postnatal Ontogenesis

**Figure 1:**
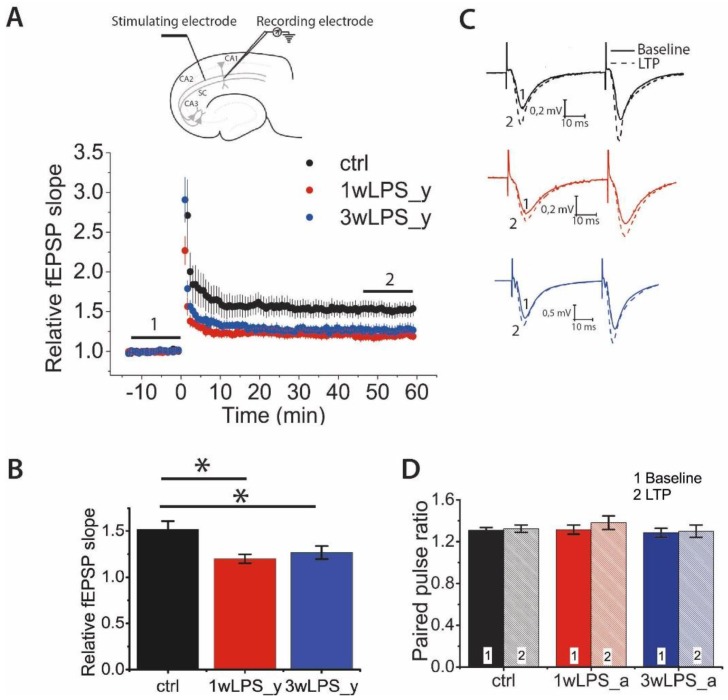
Hippocampal long-term potentiation (LTP) in young rats is weakened after the administration of lipopolysaccharide (LPS) in early postnatal ontogenesis. (**A**) The normalized field excitatory postsynaptic potentials (fEPSP) slope after theta-burst stimulation (TBS) in control (ctrl) and experimental animals injected with LPS during the first (1wLPS_y) or third (3wLPS_y) postnatal week. The insert (above) shows the positions of electrodes in the hippocampus. (**B**) Diagram illustrating the difference in LTP values between control and experimental animals (one-way ANOVA: F_2,48_ = 3.30; *p* < 0.05; the significant difference with the control group: * *p* < 0.05). (**C**) Representative examples of paired fEPSP responses before (1) and after (2) TBS in control and experimental animals. (**D**) The paired-pulse ratio (PPR) of the fEPSP amplitudes before and after the induction protocol did not change in either control or experimental rats. Repeated measures ANOVA: F_2,49_ = 2.53; *p* = 0.09 (control, n = 14; 1wLPS_y, n = 18; 3wLPS_y, n = 20).

First, we compared LTP magnitude between young rats (P21–23) in the control group and young rats in the two experimental groups in which LPS was administered, at different periods: (1) 1wLPS_y (LPS was administered three times during the first week, on P1, P3, and P5) and (2) 3wLPS_y (LPS was administered three times during the third week, on P14, P16, and P18). The LTP magnitude between these groups differed significantly (one-way ANOVA: F_2,48_ = 3.30; *p* < 0.05; Figure 1A,C). According to the Tukey post hoc test, the amplitude of LTP in the control group (1.49 ± 0.08, n = 15 slices, N = 8 rats) was twice as large as that among 1wLPS_y rats (1.25 ± 0.07, n = 16, N = 6, *p* < 0.01) and 3wLPS_y rats (1.27 ± 0.07, n = 20, N = 12, *p* < 0.05). Thus, the administration of LPS during the first or third week equally weakens LTP in young rats.

Next, we determined the locus of LTP expression. For this, we compared the paired-pulse ratio (PPR) before and after the induction of LTP (Figure 1B,D). It was assumed that if the ratios of the amplitudes of the second response to the amplitudes of the first response before and after the induction protocol were equal, then this would indicate a postsynaptic locus of expression of LTP and would be associated with the insertion of additional AMPARs into the postsynaptic membrane. A change in the paired facilitation value indicates a presynaptic locus of expression associated with a change in the probability of neurotransmitter release [43,44,45]. According to the two-way repeated measures ANOVA, there were no changes in the PPR after the induction of LTP in any group of animals (Figure 1D). Therefore, these results suggest that the postsynaptic locus of LTP expression in CA3-CA1 synapses is preserved in experimental animals.

### 2.2. Main Properties of Excitatory Synaptic Transmission in Hippocampal Pyramidal Neurons are not Altered after LPS Treatment

**Figure 2:**
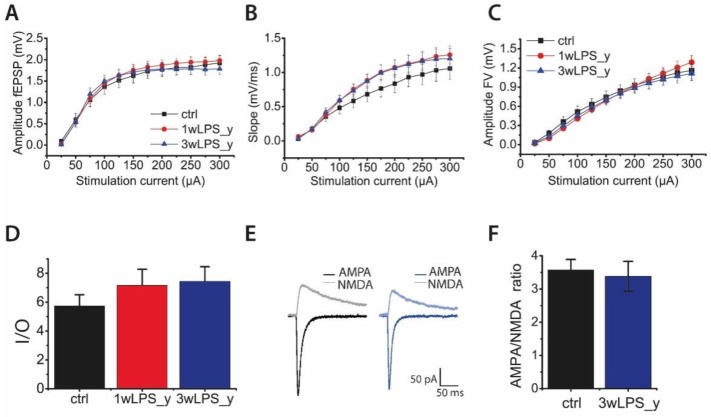
The main features of excitatory synaptic transmission in hippocampal pyramidal neurons are not altered after LPS treatment. The relationships between stimulation current and fEPSP amplitudes (**A**), slopes (**B**), and presynaptic fiber volley (FV) amplitude (**C**) recorded from the hippocampal CA1 area. (**D**) The diagram shows the maximum slope of the input/output (I/O) curve in different groups. (**E**) Representative examples of evoked EPSCs recorded at −80 mV and +40 mV from control rats and rats treated with LPS. The AMPA component was obtained by measuring the excitatory postsynaptic current (EPSC) peak amplitude at −80 mV in the presence of the GABA_A_R blockers bicuculline (10 μM) and picrotoxin (50 μM). The N-methyl-D-aspartate (NMDA) component was obtained by measuring the EPSC peak amplitude at +40 mV in the presence of gamma-aminobutyric acid type A receptor (GABAa) blockers and the AMPAR blocker, CNQX (20 μM). (**F**) The diagram illustrates the AMPA/NMDA ratio in the control and 3wLPS_y groups. Error bars indicate standard errors SE.

The decrease in the magnitude of LTP in experimental rats might be explained by alterations in the properties of synaptic transmission in the hippocampus. Therefore, we compared the input/output (I/O) parameters of excitatory synaptic transmission at the CA3-CA1 synapses. Toward this end, afferent fibers were electrically stimulated at a range of current intensities (25–300 mA), and it was found that the amplitude (Figure 2A) and the slope (Figure 2B) of the field excitatory postsynaptic potentials (fEPSPs), as well as the amplitudes of fiber volleys (FV) (Figure 2C), did not differ between the groups. Next, we determined the I/O relationship of fEPSP and FV amplitudes for each slice. The I/O relations were well fitted with a sigmoidal Gompertz function. The maximum slope of this fit can be considered as a measure of synaptic strength [46]. According to the one-way ANOVA, the maximum slope of the I/O curve was not altered in LPS-treated rats (F_2,67_ = 1.67; *p* = 0.20; Figure 2D).

Because the amplitude of fEPSP depends mostly on the activation of AMPAR-mediated conductance [47,48], potential changes in the impact of NMDAR might be undervalued. Therefore, we investigated the relative contribution of AMPAR- and NMDAR-mediated excitatory synaptic currents. Using a whole-cell voltage clamp, we recorded evoked excitatory postsynaptic currents (eEPSCs) in CA1 pyramidal cells in acute brain slices of control and 3wLPS_y animals. To evaluate the relative contribution of AMPAR- and NMDAR-mediated synaptic currents, we first applied the gamma-aminobutyric acid type A receptor (GABA_A_) antagonists bicuculline (10 μM) and picrotoxin (50 μM) and recorded AMPAR-mediated eEPSCs at −80 mV. Then, after the application of a selective AMPAR antagonist, 6,7-dinitroquinoxaline-2,3-dione (DNQX 20 μM), we recorded NMDAR-mediated eEPSCs at holding potentials of +40 mV. Next, we calculated the AMPA/NMDA amplitude ratio. We revealed no difference in these ratios between the control (3.6 ± 0.3) and 3wLPS_y (3.4 ± 0.5; *t*-test = 0.26, *p* > 0.05) groups.

**Figure 3:**
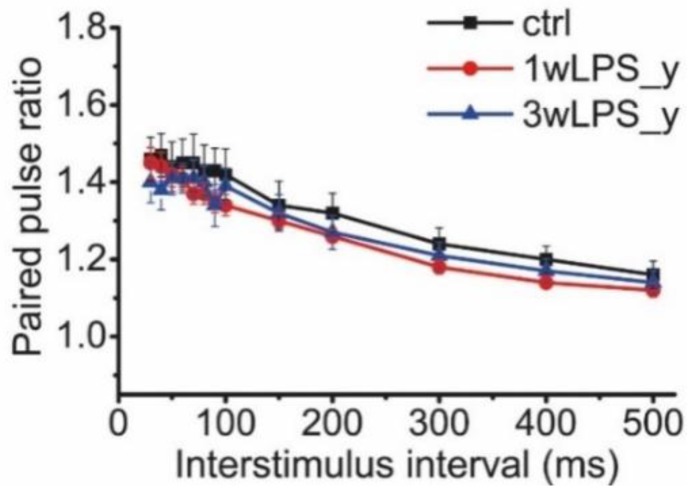
The PPRs of the fEPSPs evoked at different interstimulus intervals in the range from 30 to 500 ms in the control and experimental groups. Repeated measures ANOVA: F_24,336_ = 1.23; *p* = 0.22. Ctrl (n = 9), 1wLPS_y (n = 12), 3wLPS_y (n = 10).

Another factor that can affect LTP is a change in the probability of glutamate release in CA3-CA1 synapses. The potential alterations in the probability of neurotransmitter release might be estimated by the difference in the pair-pulse facilitation ratio (PPR) [49,50]. We compared the PPRs of the fEPSPs evoked at different interstimulus intervals in the range from 30 to 500 ms in animals of both the control and experimental groups (Figure 3). Repeated measures ANOVA suggested no difference between these groups.

Together, our data indicate that LPS treatment does not affect the main properties of glutamatergic synaptic transmission in the hippocampus, such that a decrease in LTP magnitude must be dependent on other factors.

### 2.3. Pharmacological Properties of LTP Changed in Young Rats Following LPS Treatment

In the CA1 hippocampus, the induction of LTP by the TBS protocol involves the NMDAR-dependent process [13,16,51,52,53]. The weakening of LTP caused by LPS treatment may be caused by impairment of molecular mechanisms of LTP induction due to, for example, changes in the subunit composition of NMDARs. Some previous studies have shown that the subunit composition of NMDARs may influence the sign and magnitude of long-term synaptic plasticity. The predominance of GluN2B-containing NMDARs may favor the induction of long-term depression [39,54]. To clarify whether the NMDAR-dependent mechanism of LTP induction changed or remained unaltered in experimental animals, we first induced LTP in the presence of Dizocilpine (MK-801) (10 μM), a non-competitive antagonist of NMDARs.

**Figure 4:**
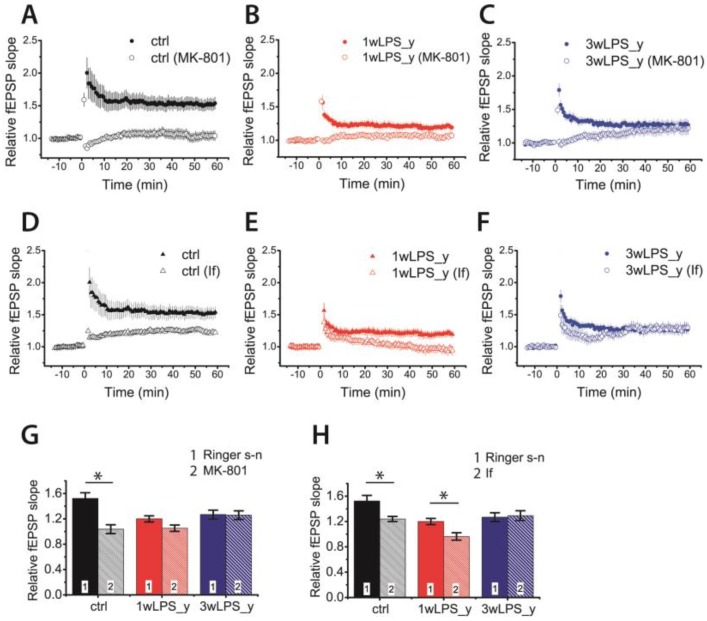
The NMDA receptor (NMDAR)-dependent mechanism of LTP induction is disrupted in the CA1 hippocampus of juvenile animals treated with LPS during the third postnatal week. (**A**–**C**) The normalized fEPSP slope in the control and experimental groups in the presence of the NMDAR blocker Dizocilpine (MK-801) (10 μM) before and after TBS. (**D**–**F**) The relative fEPSP slope in the control and experimental groups in the presence of ifenprodil (If, 3 μM), a selective GluN2B-containing NMDAR antagonist, before and after TBS. (**G**–**H**) Diagrams illustrating the magnitude of plasticity in the control and experimental groups in the presence of MK-801 or ifenprodil. Two-way ANOVA following Tukey post hoc tests were used. * *p* < 0.05.

No LTP developed in the control group in the presence of MK-801 (1.04 ± 0.07; n = 12; paired *t*-test = 4.30; *p* < 0.01; Figure 4A,B), suggesting a pure NMDA-dependent mechanism of LTP induction. The induction of LTP was also completely inhibited in slices obtained from 1wLPS_y rats (1.05 ± 0.10; n = 7). In contrast, no inhibition of LTP was observed in the 3wLPS_y group (1.27 ± 0.06, n = 20 in Ringer’s solution vs. 1.26 ± 0.08, n = 12 in the presence of MK-801). Two-way ANOVA indicated a significant interaction of two factors: a group of animal × effect of MK-801 application (F_2,76_ = 5.7, *p* < 0.01), suggesting that mechanisms of LTP induction are different in these groups. Thus, LTP induction in the 3wLPS_y group is based on a non-NMDAR Ca^2+^ source.

Next, we investigated the effect of the inhibition of GluN2B-containing NMDARs by applying a selective antagonist of GluN2B-containing NMDARs, ifenprodil (3 μM). The effect of ifenprodil was significant (two-way ANOVA: F_1,78_ = 8.0, *p* < 0.01), but the interaction between factors (group of animals and effect of ifenprodil) did not obtain a level of significance (F_2,78_ = 2.7, *p* = 0.07). The application of ifenprodil reduced LTP in the control group from 1.52 ± 0.07 (n = 15) to 1.24 ± 0.07 (n = 14, Tukey post hoc test, *p* < 0.05). The diminished LTP in the 1wLPS_y group (1.20 ± 0.06, n = 16) was completely abolished (0.96 ± 0.08, n = 10, *p* = 0.03). In agreement with our previous results about the NMDA-independent mechanism of LTP induction in the 3wLPS_y group, the application of ifenprodil did not affect the amplitude of LTP in this group (1.27 ± 0.06, n = 20 vs. 1.29 ± 0.09, n = 9, *p* = 0.81).

**Figure 5:**
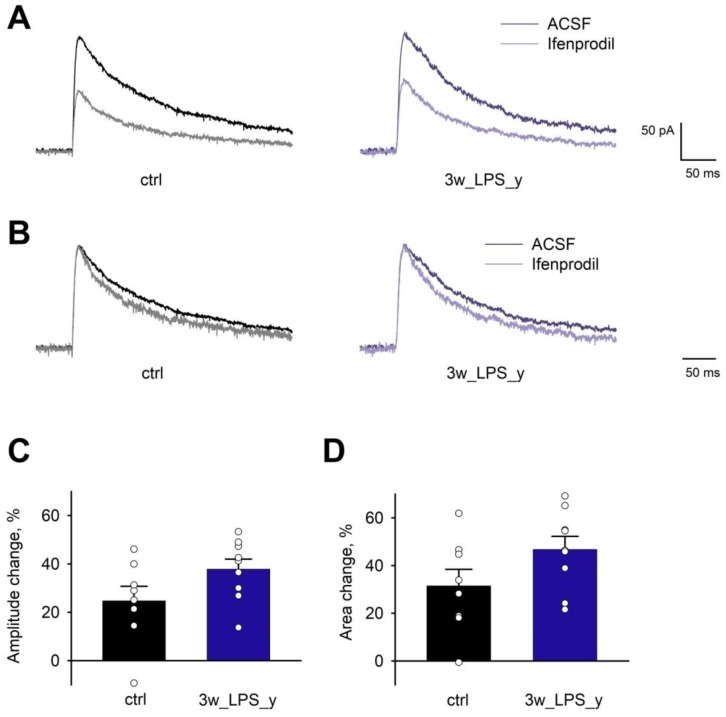
The effect of ifenprodil on eEPSCs in CA1 pyramidal neurons. (**A**) Representative examples of evoked NMDAR-mediated EPSCs recorded at +40 mV in the presence of bicuculline (10 μM), picrotoxin (50 μM), and CNQX (20 μM) in the control and 3wLPS_y groups. (**B**) The same traces as in (A) normalized by amplitude. Note that ifenprodil decreases tau decay. (**C**,**D**) The effect of ifenprodil on amplitude and area under the curve of NMDAR-mediated eEPSC. Dot points represent data from individual cells. Error bars indicate SE.

Next, we compared the properties of synaptic NMDAR-mediated currents in CA1 hippocampal pyramidal neurons using voltage-clamp, whole-cell recordings in slices obtained from 3wLPS_y, and control animals (Figure 5). The GluN2A-containing NMDARs exhibited faster kinetics than the GluN2B-containing receptors [55]. In our preparation, we found that the decay kinetics of the NMDAR-mediated synaptic currents were similar in the control and LPS-treated rats (weighted time constant: 212 ± 14 ms, N = 6 rats, n = 8 neurons vs. 176 ± 11 ms, N = 7, n = 9, accordingly; *t*-test = 2.05, *p* > 0.05). We also tested the blocking effect of ifenprodil (3 μM) and did not observe any differences in the percentage of the blocking of the EPSC amplitude (control: 25 ± 6% vs. 3wLPS_y: 38 ± 4%; t = 1.82; *p* > 0.05) or the area under a curve (control: 31 ± 7% vs. 3wLPS_y: 47 ± 5%; t = 1.74; *p* > 0.05) of NMDAR-mediated evoked EPSCs between control and LPS-injected animals. Together, these results indicate that a disturbance in the LTP induction mechanism in LPS-treated animals cannot be explained by changes in the subunit composition of synaptic NMDARs.

**Figure 6:**
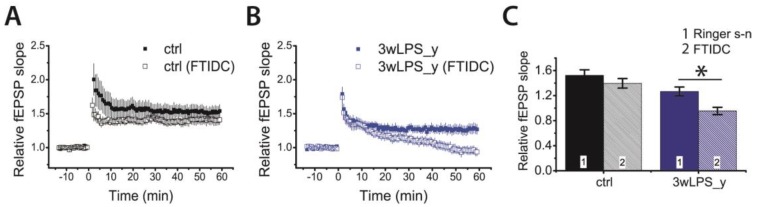
Metabotropic glutamate receptor (mGluR)-dependent form of LTP presents in the hippocampal CA1 area of juvenile rats administered with LPS during the third postnatal week. Diagrams showing the normalized slope of fEPSP in the control (**A**) and experimental (**B**) groups. Note that FTIDC (4-[1-(2-fluoropyridin-3-yl)-5-methyltriazol-4-yl]-N-methylN-propan-2-yl-3,6dihydro-2H-pyridine-1-carboxamide) (5 μM) affected LTP only in the 3wLPS_y group. (**C**) Diagram illustrating the effect of FTIDS on LTP values in different groups of rats, respectively * *p* < 0.05 (*t*-test).

In the presence of MK-801, the amplitude of fEPSPs in the 3wLPS_y group slowly increased after TBS and reached a maximum after 30–60 min (Figure 4C). Such a change in the amplitude of fEPSPs after the induction protocol is a characteristic of mGluR-dependent forms of LTP [33,42,56]. Therefore, we tested the effect of a selective mGluR1 antagonist, FTIDC (4-[1-(2-fluoropyridin-3-yl)-5-methyltriazol-4-yl]-N-methylN-propan-2-yl-3,6dihydro-2H-pyridine-1-carboxamide) (5 μM) [57], on LTP induction in different groups. Its application did not significantly affect LTP magnitude in the control group (1.52 ± 0.08, n = 15 vs. FTIDC: 1.40 ± 0.08, n = 13, *p* = 0.26, Figure 6A,C); however, it abolished LTP induction in the 3wLPS_y group (1.27 ± 0.06, n = 20 vs. FTIDC: 0.95 ± 0.07, n = 17, *p* < 0.01, Figure 6B,C)

These results suggest that the group I mGluR-dependent form of LTP is predominant in CA3-CA1 synapses following LPS treatment during the third week.

### 2.4. LPS Administration in Early Postnatal Ontogenesis Does Not Have a Delayed Impact on Synaptic Plasticity in the Hippocampus of Adolescent Rats

**Figure 7:**
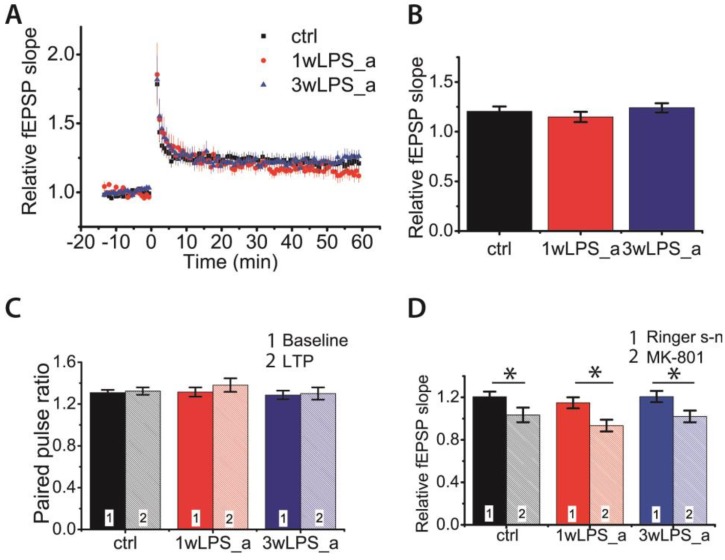
LTP properties are not disturbed in adolescent rats administered with LPS during the first postnatal week (1wLPS_a) or third postnatal week (3wLPS_a). (**A**) Diagram showing the normalized slope of fEPSP in the control (ctrl) and experimental groups (1wLPS_a and 3wLPS_a) before and after TBS. (**B**) Diagram illustrating average LTP values in these groups. One-way ANOVA: F_2;48_ = 0.27; *p* = 0.77. (**C**) Diagram showing the PPRs of fEPSPs before (baseline) and after (LTP) TBS in different groups. (**D**) Diagram illustrating the effect of MK-801 (10 μM) on LTP magnitude in the control (ctrl) and experimental (1wLPS_a; 3wLPS_a) groups * *p* < 0.05.

Our next question was how long the effects of LPS treatment on synaptic plasticity last. We investigated the properties of CA1 hippocampal LTP in P51-55 rats that underwent LPS treatment during the first (1wLPS_a, n = 10 slices, N = 8 animals) or third (3wLPS_a, n = 12, N = 8 animals) postnatal weeks with control rats (n = 29, N = 19 animals) of the same age. One-way ANOVA did not reveal significant differences in the LTP magnitude (F_2,48_ = 0.27; *p* = 0.77; Figure 7A,B).

Next, we checked whether the locus of LTP expression had changed. We compared the PPRs of fEPSP amplitudes before and after the induction of LTP. The repeated measures ANOVA revealed no changes in PPRs after the induction of LTP in any group of animals (F_2,48_ = 0.58; *p* = 0.56; Figure 7C), suggesting the postsynaptic locus of LTP expression in all tested groups. We also examined whether the NMDAR-dependent mechanism of LTP induction was preserved in the experimental group. We found that the application of MK-801 (10 μM) in all groups completely prevented synaptic plasticity (control group: 0.98 ± 0.04; n = 10; 1wLPS_a: 0.93 ± 0.06; n = 11; 3wLPS_a 1.02 ± 0.05; n = 9; Figure 7D). Together, these results suggest that LPS treatment in early ontogenesis does not affect the properties of synaptic plasticity in P51-55 rats.

### 2.5. LPS Administration in Early Ontogenesis Induces Some Changes in the Behavior of Young and Adult Rats

**Figure 8:**
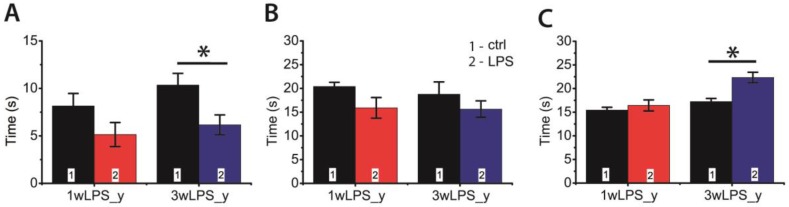
The behavior of P23-25 rats in the open field-test, which were treated with LPS during the first (1wLPS_y) or third (3wLPS_y) weeks. (**A**) Total time of hole exploration (effect of treatment: F_1,50_ = 11.2; *p* = 0.002); (**B**) Time of locomotion (effect of treatment: F_1,54_ = 4.01; *p* = 0.05); (**C**) Duration of movement on the spot (interaction time × treatment: F_1,51_ = 5.3; *p* = 0.03; time: F_1,51_ = 17.9; *p* < 0.001; treatment: F_1,51_ = 11.1; *p* = 0.002). * *p* < 0.05 (Tukey post hoc test).

**Figure 9:**
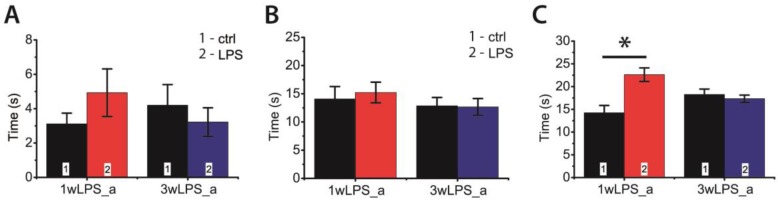
The behavior of P90 rats in the open field-test, which were treated with LPS during the first (1wLPS_a) or third (3wLPS_a) weeks. (**A**) Total time of hole exploration (interaction time × treatment: F_1,45_ = 1.77; *p* = 0.19; time: F_1,45_ = 0.09; *p* = 0.77; treatment: F_1,45_ = 0.16; *p* = 0.69); (**B**) Time of locomotion (interaction time × treatment: F_1,45_ = 0.14; *p* = 0.71; time: F_1,45_ = 1.14; *p* = 0.29; treatment: F_1,45_ = 0.08; *p* = 0.78); (**C**) Duration of movement on place (interaction time × treatment: F_1,45_ = 13.21; *p* < 0.01; time: F_1,45_ = 0.25; *p* = 0.62; treatment: F_1,45_ = 8.41; *p* < 0.01). * *p* < 0.05 (Tukey post hoc test).

Next, we investigated whether LPS administration in early ontogenesis induces some changes in the behavior of young and adult rats. We performed an open field test in P23–25 and P90 rats. In young animals, we observed some disturbances in investigative behavior that manifested as a decrease in the time of hole exploration (Figure 8A). In addition, the LPS-treated animals exhibited reduced motor activity, showing a shorter time of locomotion at the new location; the time spent moving on the spot was larger (Figure 8B,C). No disruption of investigative behavior was found in P90 rats (Figure 9A). On the other hand, the administration of LPS during the first postnatal week affected motor activity in adult rats, increasing the time spent moving on the spot (Figure 9C), but time of locomotion did not differ with the control groups (Figure 9B).

**Figure 10:**
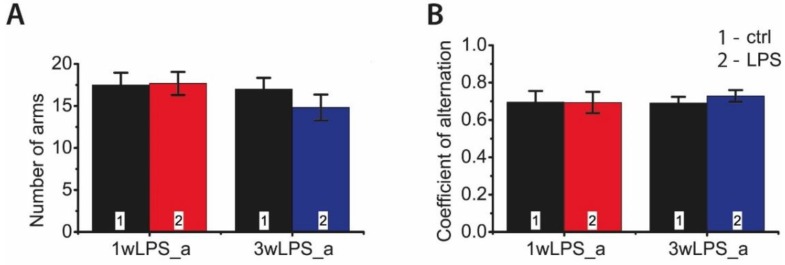
The behavior of P90 rats in the Y-maze test, which were treated with LPS in early postnatal ontogenesis. (**A**) Number of visited arms (interaction time × treatment: F_1,38_ = 0.66; *p* = 0.42; time: F_1,38_ = 1.34; *p* = 0.25; treatment: F_1,38_ = 0.49; *p* = 0.49). (**B**) Coefficient of alternation (interaction time × treatment: F_1,38_ = 0.19; *p* = 0.67; time: F_1,38_ = 0.11; *p* = 0.74; treatment: F_1,38_ = 0.16; *p* = 0.70).

Animals treated with LPS, during either the first or the third postnatal week, demonstrated no spatial memory impairments at P90 in the Y-maze test (Figure 10). The coefficients of alternation did not differ in the groups, nor were any differences observed in total motor activity in the Y-maze test (total number of visited arms).

## 3. Discussion

In this study, we aimed to investigate and compare the short- and long-term effects of the repetitive administration of LPS in low doses during the first or third postnatal weeks on synaptic plasticity. These terms of administration were chosen since the synapses are immature in the first week and maturation occurs during the third week [41,58,59]. We revealed that LTP induction in the hippocampus was similarly weakened in both experimental groups of young rats independently of time of LPS administration. However, the mechanisms of LTP induction differed between the experimental groups. The administration of LPS during the third week completely suppressed the NMDAR-dependent form of LTP, and LTP switched to the mGluR1-dependent form; while in rats treated with LPS during the first week, a typical postsynaptic NMDA-dependent form of LTP was revealed. These impairments in synaptic plasticity were accompanied by some disorders in investigative and motor activity in the open field test. However, our results indicate that these impairments of synaptic plasticity and behavior are temporary. In adolescent rats, we observed no difference in LTP properties between the control and experimental groups. The parameters of investigative and motor activity in the control and experimental groups of adult rats were similar.

LPS may affect synaptic plasticity through different mechanisms. Previous studies have shown that LTP magnitude in hippocampal slices is acutely reduced after in vitro application of LPS [19,60], as well as 3 h after in vivo administration of a high dose of LPS (0.83 mg/kg) [4]; while a lower dose of LPS (0.33 mg/kg) does not affect LTP magnitude [4]. The authors suggested that high doses of LPS potently stimulate the release of IL-1 and TNF, which suppress LTP induction by inhibiting either presynaptic or postsynaptic calcium channels [19]. The alternative mechanism suggested by Iwai et al. [60] is that induced IL-1b inhibits LTP via the activation of p38 MAPK and c-Jun N-terminal kinases (JNK) [61], and that these signal pathways inhibit AMPAR trafficking [62], which is an important mechanism of LTP induction [63]. In other studies, the repetitive administration of LPS in low doses induced a delayed decrease in LTP magnitude [8] and learning and memory deficits in rats [64]. The authors have shown that the gene expression levels of neurotrophic factor, BDNF, and its receptor, TrkB, were significantly decreased by LPS treatment, and glutamatergic transmission was attenuated in LPS-treated rats as well [8,64].

In our study, we focused on the properties of glutamatergic neurotransmission. First, we investigated the properties of AMPAR-mediated transmission. Because the contribution of NMDARs to fEPSP is relatively small, any differences in I/O curves are dependent specifically on AMPAR-mediated input [48]. All I/O parameters, including the amplitude and slope of the fEPSPs and the amplitudes of FV, did not differ between the groups of animals. Thus, we could not confirm significant changes in AMPAR-mediated excitatory neurotransmission in the CA1 hippocampus.

NMDARs are closely involved with synaptic plasticity, as well as with learning and memory [34,38]. Selective loss of NMDAR subunits in CA1 hippocampal neurons was shown in the sepsis model using a high dose of LPS [65]. Chronic brain inflammation induced by chronic infusion of LPS into the fourth ventricle also causes a reduction in GluN2A and GluN2B subunits of NMDARs [66]. However, a single injection of LPS in a relatively low dose (100 μg/kg) also produces acute and long-term changes in the subunit composition of NMDARs, including a decrease and an increase of different subunits at different time points [2]. The third postnatal week is a critical period for the maturation of synaptic functions and NMDARs in the rat hippocampus [41,67]. Changes in the AMPAR/NMDAR ratio are commonly associated with postsynaptic maturation synapses [68,69]. Therefore, we assumed that the loss of the NMDAR-dependent form of LTP was associated with some disturbances in NMDAR properties. Yet, we found no difference in the AMPAR/NMDAR ratios in eEPSCs between the control and 3wLPS_y groups, suggesting, together with I/O data, that the NMDAR-mediated current is not diminished in experimental animals. We observed no difference in tau-decay of NMDAR-mediated eEPSCs or in the effects of subunit-selective NMDAR antagonist ifenprodil on postsynaptic currents, suggesting that the subunit composition of synaptic NMDAR was not disturbed by LPS administration. Thus, our hypothesis about the reduced NMDAR-mediated synaptic current was not directly confirmed.

Our finding of a temporary switch from the NMDAR-dependent form of LTP to the mGluR1-dependent form in 3wLPS_y rats suggests that NMDARs, as calcium sources and intracellular calcium sensors, are dissociated, and that the normal LTP signal transduction mechanism is disturbed. LTP can involve several mechanism [14,18,30]. Clarke R. Raymond showed that moderate stimulation, similar to that used in our LTP induction protocol, typically induces a form of LTP dependent on the activation of NMDARs but uniquely sensitive to the IP3 receptor (IP3R) blockade. IP3Rs are primarily activated by Ins(1,4,5)P3 generated by phospholipase C (PLC)-linked group I mGluRs [18]. The coexistence of distinct activity-dependent systems of synaptic plasticity based on discrete Ca^2+^ sources, such as NMDARs and mGluRs, has been recently described in the same synapses [33]. In our experiments, LTP induction did not require the activity of NMDARs because it was preserved, even when the NMDARs were blocked with MK-801. This form of plasticity resembles the LTP observed in the hippocampal slices of rats one day after pentylenetetrazole-induced status epilepticus [42].

The over-activation of group I mGluRs following in vivo systemic LPS or IL-1β was shown for neonatal and adult rodent cortical neurons [70]. Although the authors did not detect changes in the expression of mGluRs, they did demonstrate that exposure to LPS or IL-1β leads to G protein-coupled receptor kinase 2 (GRK2) down-regulation, leading to over-activation of group I mGluRs and subsequently to sustained calcium mobilization [70]. In normal conditions, GRK2 leads to rapid desensitization of mGlu1/5, limiting the PLCβ1-mediated calcium release from endogenous stores [71]; a reduced content of GRK2 prevents the complete desensitization of mGlu1/5 and allows a more prolonged calcium release from endogenous stores [70]. We suggest that this enhanced calcium mobilization may lead to the appearance of a new form of LTP.

It is important to note that synaptic plasticity disturbances were detected only in young rats. In adolescent rats, we revealed no differences in the properties of LTP in control and experimental rats, although some very mild behavioral disturbances were observed. Our results suggest that synaptic functions disturbed by bacterial infections in early childhood can almost be completely restored. However, previous studies showed persistent effects of postnatal systemic inflammatory challenges on escape learning in the footshock-elicited active avoidance and water maze paradigms [10]; other researchers also observed long-lasting changes in NMDAR mRNA expression, which were also associated with behavioral deficits [2]. Differences in these behavioral results may be due to differences in experimental conditions. Control and experimental animals showed no difference in “easy” behavioral tests, such as open field or Y-maze tests; however, in more stressful tests, such as the water maze, differences between groups were observed, suggesting that animals exposed to LPS in early life periods may display altered susceptibility to stress in later life [2,10].

## 4. Materials and Methods

### 4.1. Animals

Wistar rats were used in this study. All rats were kept under standard conditions at room temperature, with free access to water and food. All experiments were carried out in accordance with the Guidelines on the Treatment of Laboratory Animals effective at the Sechenov Institute of Evolutionary Physiology and Biochemistry of the Russian Academy of Sciences; these guidelines also comply with Russian and international standards. The animal experiments in this study were approved by the Sechenov Institute of Evolutionary Physiology and the Biochemistry Ethics Committee. All efforts were made to minimize the number and degree of suffering of the animals used.

Repeated administration of bacterial LPS was used to model the bacterial infection. All experiments used LPS derived from *Escherichia coli*, serotype 055:B5 (Sigma Aldrich, St. Louis MO, USA, Cat # F8666) at a concentration of 25 µg/kg.

Two experimental groups of animals were formed. LPS was injected intraperitoneally for 3 days either during the first week of life—on postnatal day P1, P3, and P5 (the first experimental group, N = 35 animals)—or during the third week of life—on postnatal day P14, P16, and P18 (the second experimental group, N = 41 animals). Control animals either received the equivalent volume of pyrogen-free saline at the same age (N = 34 animals) or received no treatment (N = 42 animals).

### 4.2. Hippocampal Brain Slice Preparation

Male juvenile (P21–23) and adolescent (P51–55) Wistar rats were used in the electrophysiological experiments. The rats were first deeply anesthetized with isoflurane and then decapitated, after which their brains were quickly removed and placed into an ice-cold (4 °C), oxygenated (95% O2: 5% CO2), artificial cerebrospinal fluid (ACSF) containing (in mM): 126 NaCl, 24 NaHCO3, 2.5 KCl, 2 CaCl2, 1.25 NaH2PO4, 1 MgSO4, and 10 dextrose. Horizontal brain slices (400 μm) containing the dorsal hippocampus were prepared with a vibratome (HM 650V, Microm International, Germany). Afterward, slices were allowed to recover at 35 °C in oxygenated ACSF for 1 h before electrophysiological experiments.

### 4.3. Electrophysiology

For the electrophysiological studies, the hippocampal slices were transferred to a recording chamber maintained at room temperature and continuously perfused with ACSF. Extracellular field excitatory postsynaptic potentials (fEPSPs) were registered from the CA1 stratum radiatum using glass microelectrodes (0.2–0.8 MΩ). A bipolar, twisted, stimulating electrode made of insulated nichrome wire (0.1 mm in diameter) was placed in the CA1 area of the hippocampal slice to stimulate the Schaffer collateral fibers. The stimulation was performed with paired pulses with an interstimulus interval of 50 ms every 20 s. A baseline was recorded at a stimulus intensity that gave 40–50% of the amplitude at which the population spike appeared. LTP was induced only after obtaining stable fEPSPs for at least 20 min. LTP was induced using theta-burst stimulation (TBS, 5 bursts of 5 100-Hz pulses, with a 200-ms interval between bursts, applied 5 times every 10 s). After LTP induction, the fEPSPs were recorded for 60 min. The LTP value was determined quantitatively as the ratio of the average fEPSP slopes recorded from 50 to 60 min after LTP induction to the average fEPSP slopes recorded immediately before LTP induction.

### 4.4. Patch-Clamp Experiments

Whole-cell recordings were obtained from visually identified CA1 hippocampal pyramidal neurons by using a Zeiss Axioscop 2 microscope (Zeiss, Oberkochen, Germany) equipped with differential interference contrast optics and a video camera (WAT-127LH, Watec Inc., Newburgh, NY, USA, or PointGrey Grasshopper3 GS3-U3-23S6M-C, FLIR Integrated Imaging Solutions Inc., USA). Patch pipettes with a 2–5-MΩ tip resistance were pulled from borosilicate-filamented glass capillaries (World Precision Instruments, Friedberg, Germany) using a P-1000 Micropipette Puller (Sutter Instrument, Novato, CA, USA). The intracellular patch pipette solution for whole-cell recordings contained (in mM): 127 CsMeS, 10 NaCl, 5 EGTA, 10 HEPES, 6 QX314, 4 ATP-Mg, and 0.3 GTP (with pH adjusted to 7.25 with CsOH). Signals were recorded using a Model 2400 patch-clamp amplifier (AM-Systems, Sequim, WA, USA), an NI USB-6343 analog-to-digital (A/D) converter (National Instruments, Austin, TX, USA), and WinWCP 5 software, or using an EPC-8 amplifier (HEKA Elektronik, Lambrecht, Germany) and PatchMaster software by the same manufacturer. Access resistance typically was 10–15 MΩ and remained stable during the experiments (< 30% increase) for all cells included in the analysis. The synaptic responses were evoked extracellularly. The bipolar, twisted, stimulating electrode was placed at a distance of 100–200 μm from the recorded neuron.

The recordings of AMPAR-mediated postsynaptic currents were performed in the presence of GABAaR blockers (10 μM bicuculline and 50 μM picrotoxin) using a whole-cell patch clamp with the holding potential set at −80 mV. NMDAR-mediated postsynaptic currents were recorded in the presence of GABAaR blockers and the AMPAR antagonist DNQX (20 μM) at the holding potential of +40 mV. The possible changes in NMDAR subunit composition were assessed using a blocker of GluN2B-containing NMDARs, ifenprodil (3 μM). The weighted decay time constants for each recorded cell were obtained by (two-exponential) fitting 20–80% of the decay of the averaged NMDAR-mediated postsynaptic current. Data were analyzed with Clampfit 10.0 software (Molecular Devices Corporation, USA).

### 4.5. Drugs

Dizocilpine (MK-801, Alomone Labs, Israel, cat # M-230, 10 µM), a non-competitive antagonist of NMDAR, 4-[1-(2-fluoropyridin-3-yl)-5-methyltriazol-4-yl]-N-methylN-propan-2-yl-3,6dihydro-2H-pyridine-1-carboxamide (FTIDC, Alomone Labs, cat # F-190, 5 µM), a potent and selective antagonist of mGlu1Rs, and 4-[2-(4-Benzylpiperidin-1-yl)-1-hydroxypropyl]phenol (ifenprodil, Alomone Labs, cat # I-105, 3 µM), a NMDAR antagonist that selectively inhibits receptors containing the NR2B subunit, were used for the electrophysiology experiments and were diluted in distilled water and then bath-applied.

### 4.6. Behavioral Testing

#### 4.6.1. Open Field Test

The open field test was performed to evaluate motor and explorative activity [72]. The rats were placed in the center of a round arena for 3 min. The arena was 1-m in diameter, with a wall height of 30 cm, and the illumination of the field was 8 Lx. The field has holes with a diameter of 4 cm in the floor; thus, the apparatus was built to perform both open field test and a hole board paradigm. The video data were analyzed offline using Pole 4 software (Institute of Experimental Medicine, St. Petersburg, Russia). The distance traveled was calculated. The time and number of the following behavioral patterns were calculated: climbing, sniffing, and exploration of the holes (i.e., a measure of the level of explorative activity), and the time of locomotion and of movement on a place.

#### 4.6.2. Y-Maze Spontaneous Alternation Test

This test was used for the evaluation of spatial working memory [73]. The Y-maze consisted of three arms (each 50 × 10 cm), with opaque walls 30-cm high. The rats were placed in the center of the 3-arm Y-maze and allowed to move freely in the maze for 8 min. The sequence of entries into the arms was estimated using the coefficient of alternation (CA). The CA parameter was calculated as follows: CA = N_right_/N_total_, where N_right_ means the number of right entries into a new arm (right entry means entry to the arm, different from arms of two previous entries; for instance, 1-2-3 or 2-3-1 or 1-3-2), and N_total_ means the total number of entries. The CA was considered as an index of operative spatial memory.

### 4.7. Data Analysis and Statistics

Data are expressed as the mean with the standard error of the mean. *N* corresponds to the number of animals used, *n* corresponds to the number of experiments (the number of slices or the number of neurons in electrophysiological experiments). Statistical analysis was performed with the Statistica 8.0 (StatSoft, USA) and OriginPro 8 (OriginLab Corporation, USA) software. Dixon’s Q test for a single outlier (at 95% confidence) or Iglewicz and Hoaglin’s robust test for multiple outliers (two-sided test) was used to reject outliers. The normality of the sample data was evaluated using the Kolmogorov–Smirnov test. The equality of variance was assessed with the Levene median test. For data that had a normal distribution and passed the equal variance test, statistical significance was assessed via a Student’s *t*-test or one-way or two-way ANOVA, where appropriate. Tukey test was used for post hoc comparison. A *p*-value of <0.05 was considered statistically significant.

## Abbreviations

DNQX	6,7-dinitroquinoxaline-2,3-dione
eEPSC	evoked excitatory postsynaptic current
fEPSP	field excitatory postsynaptic potential
FV	fiber volley
FTIDC	4-[1-(2-fluoropyridin-3-yl)-5-methyltriazol-4-yl]-N-methylN-propan-2-yl-3,6dihydro-2H-pyridine-1-carboxamide
IL-1β	interleukin-1β
IL-6	interleukin 6
LPS	lipopolysaccharide
LTP	long-term potentiation
NMDA	N-methyl-D-aspartate
NMDAR	NMDA receptor
PPR	paired-pulse ratio
TBS	theta-burst stimulation
TNF-α	tumor necrosis factor alpha
